# Supplementary material for: A new mRNA antigen vaccine induces potent B and T cell responses and in vivo protection against SARS-CoV-2
Source: NPJ Vaccines. 2026 Mar 24;11:100. doi: 10.1038/s41541-026-01421-z (PMC13172340; doi:10.1038/s41541-026-01421-z)
Supplement: Supplementary file 1 — Supplementary information [file 41541_2026_1421_MOESM1_ESM.pdf]

Supplementary Fig 1

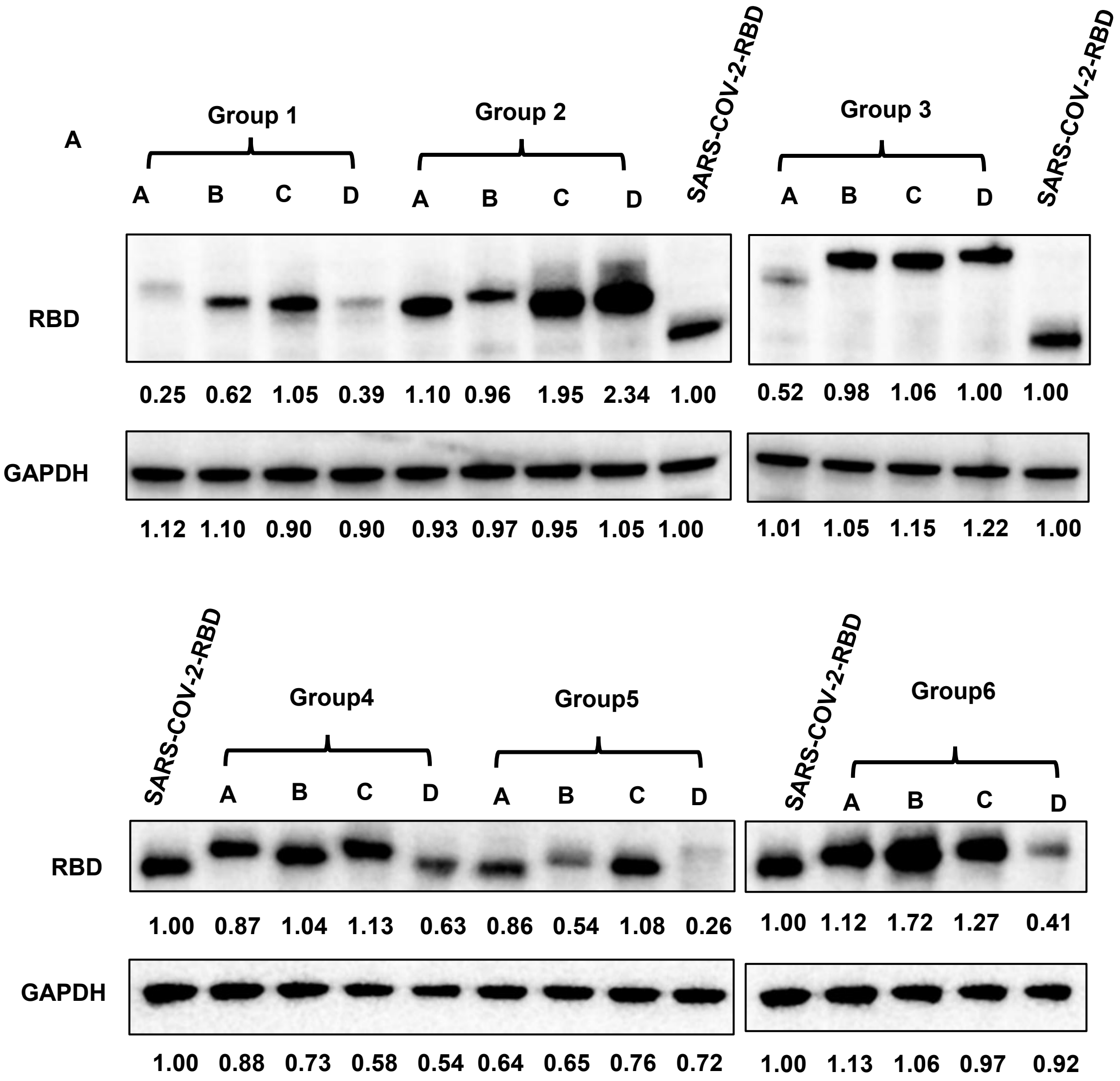

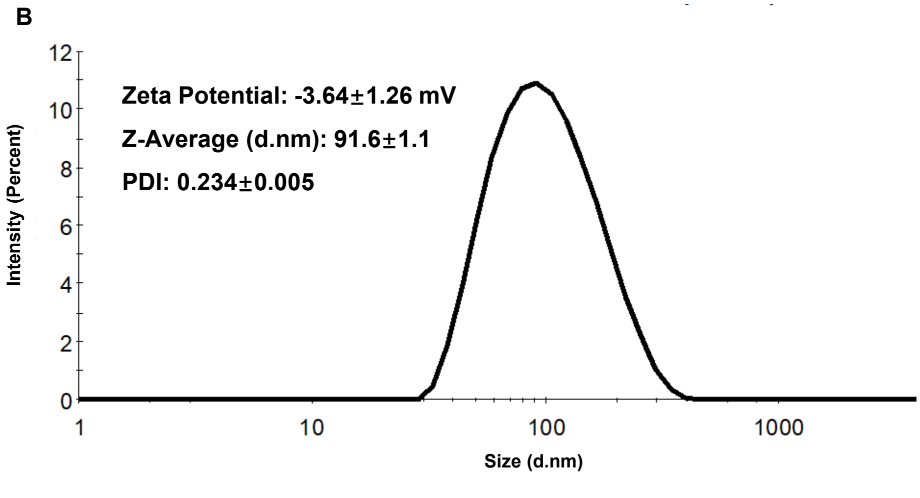

Supplementary Fig 2

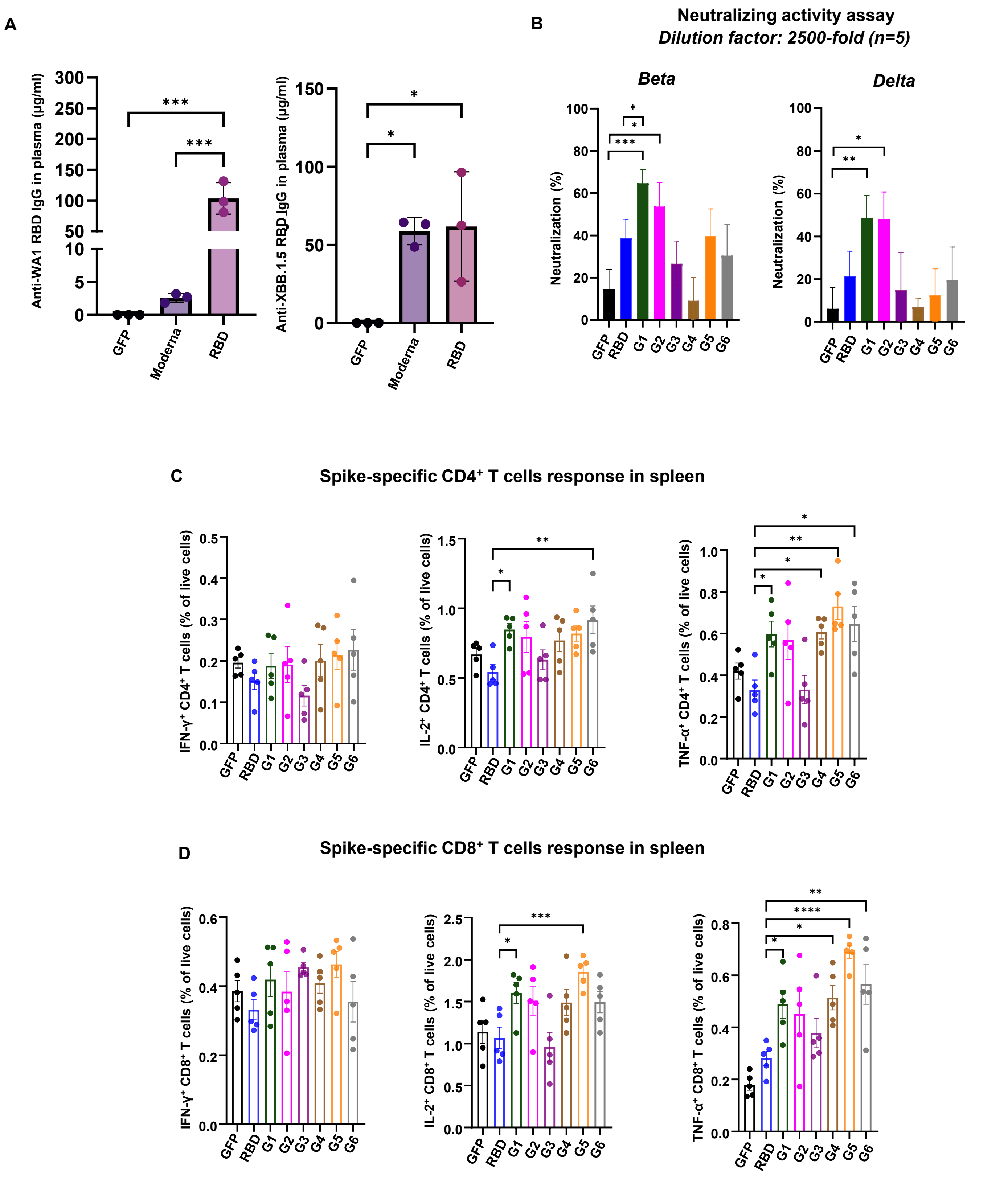

Supplementary Fig 3

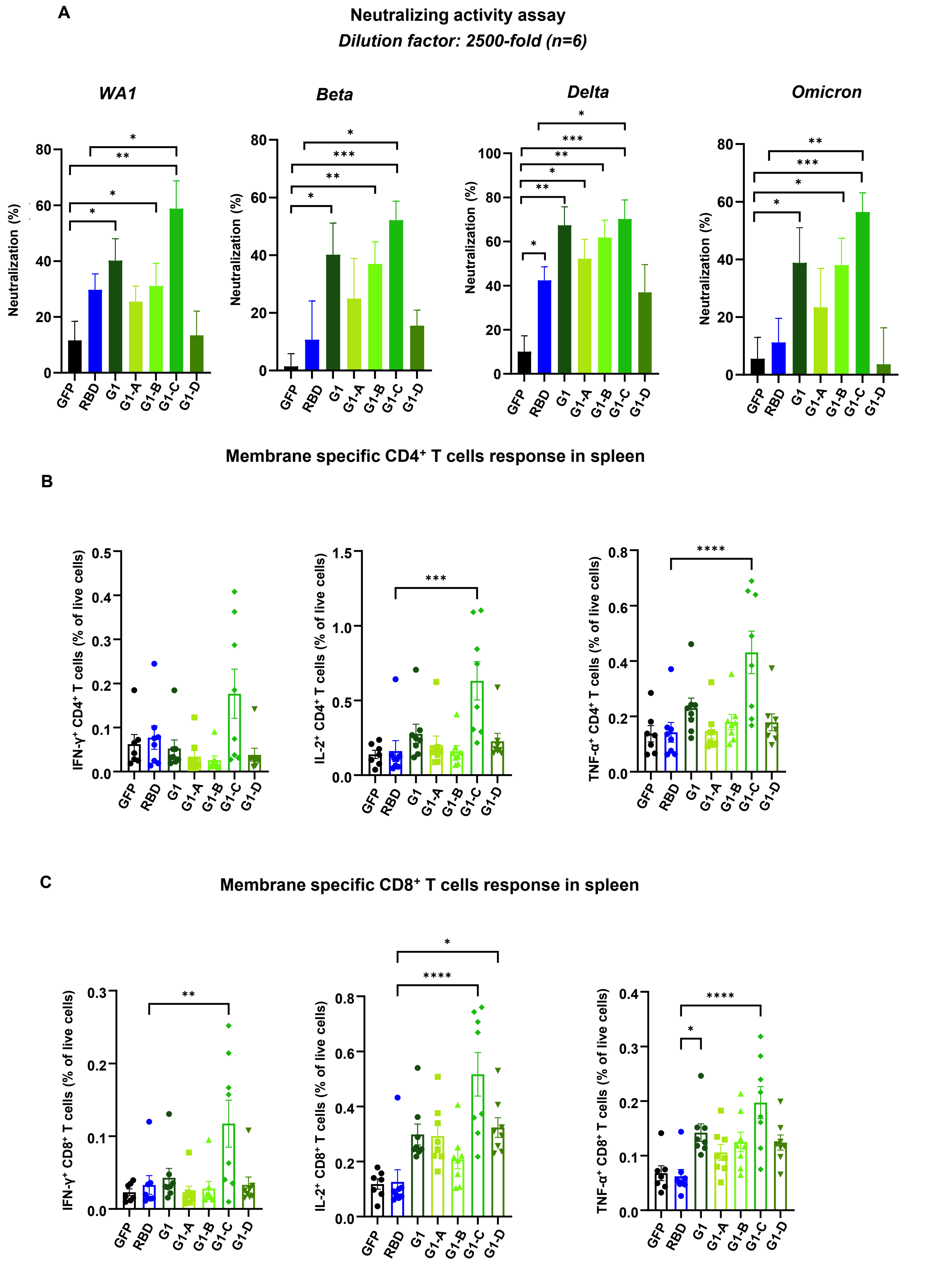

Supplementary Fig 4

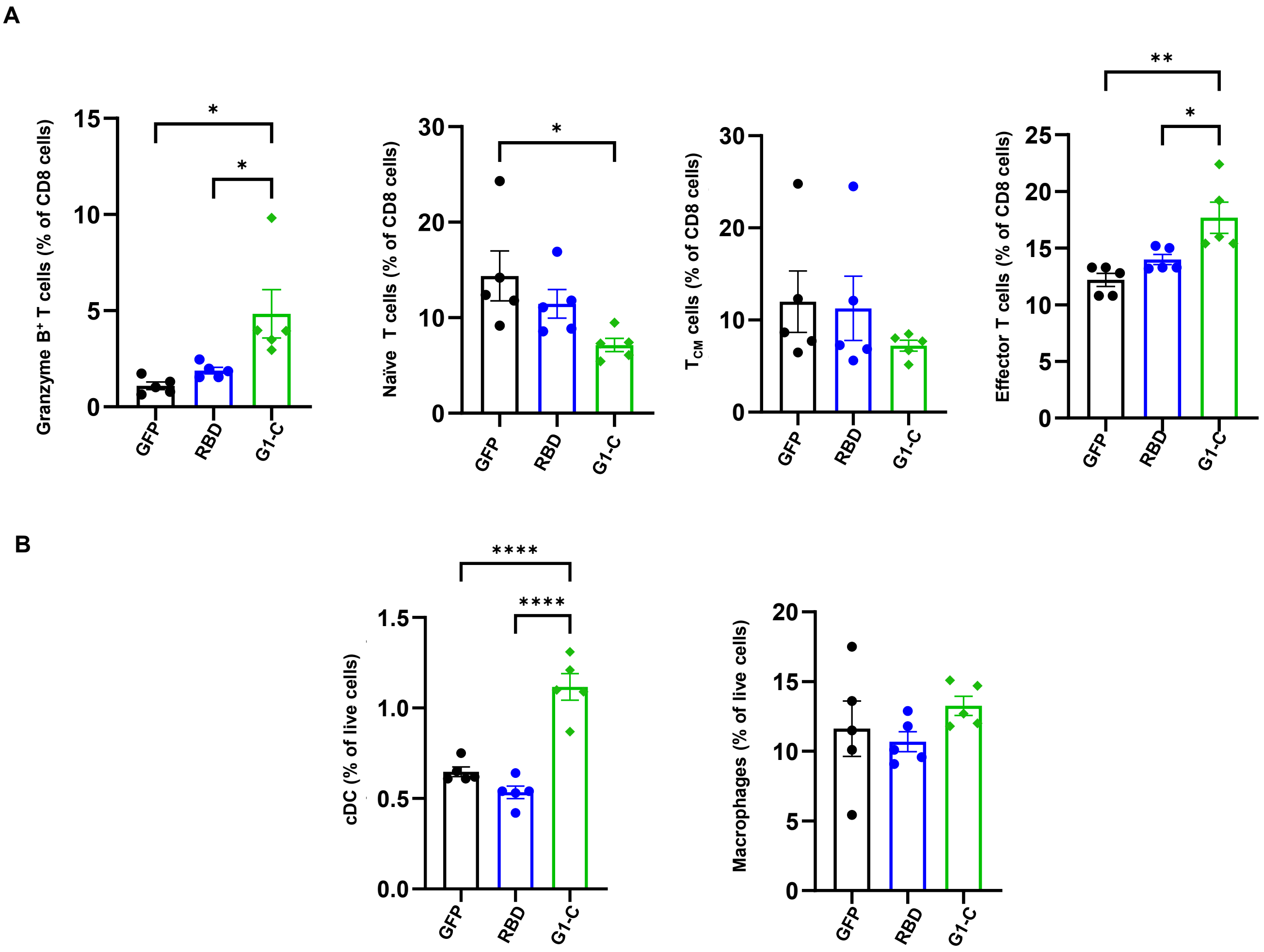

Supplementary Fig 5

A

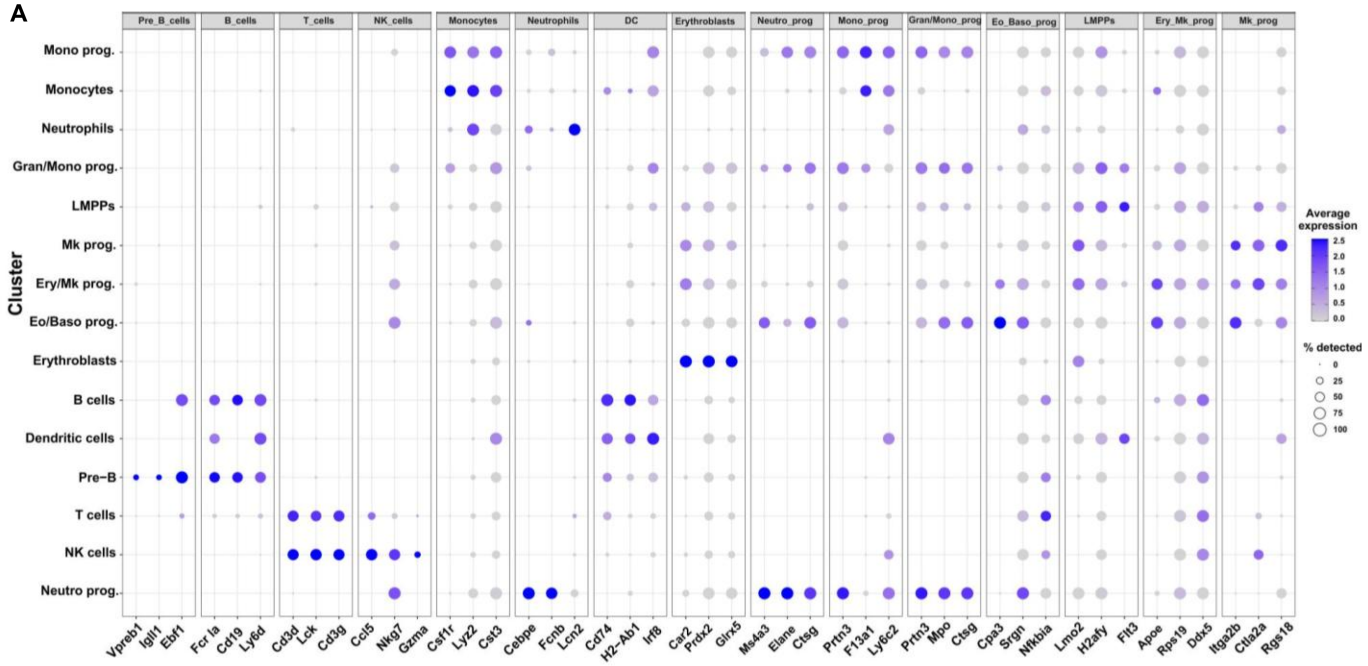

B

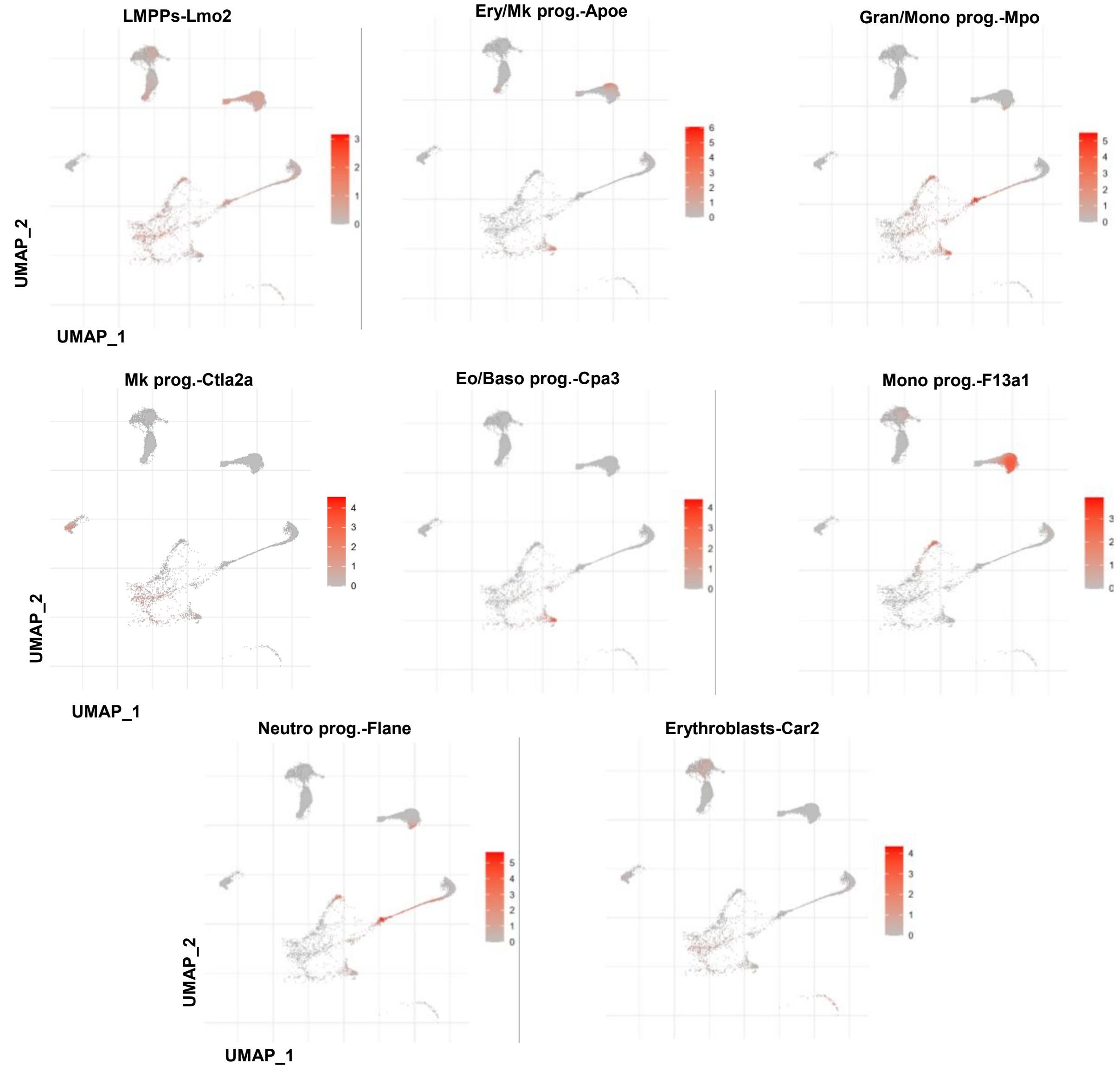

Supplementary Fig 6

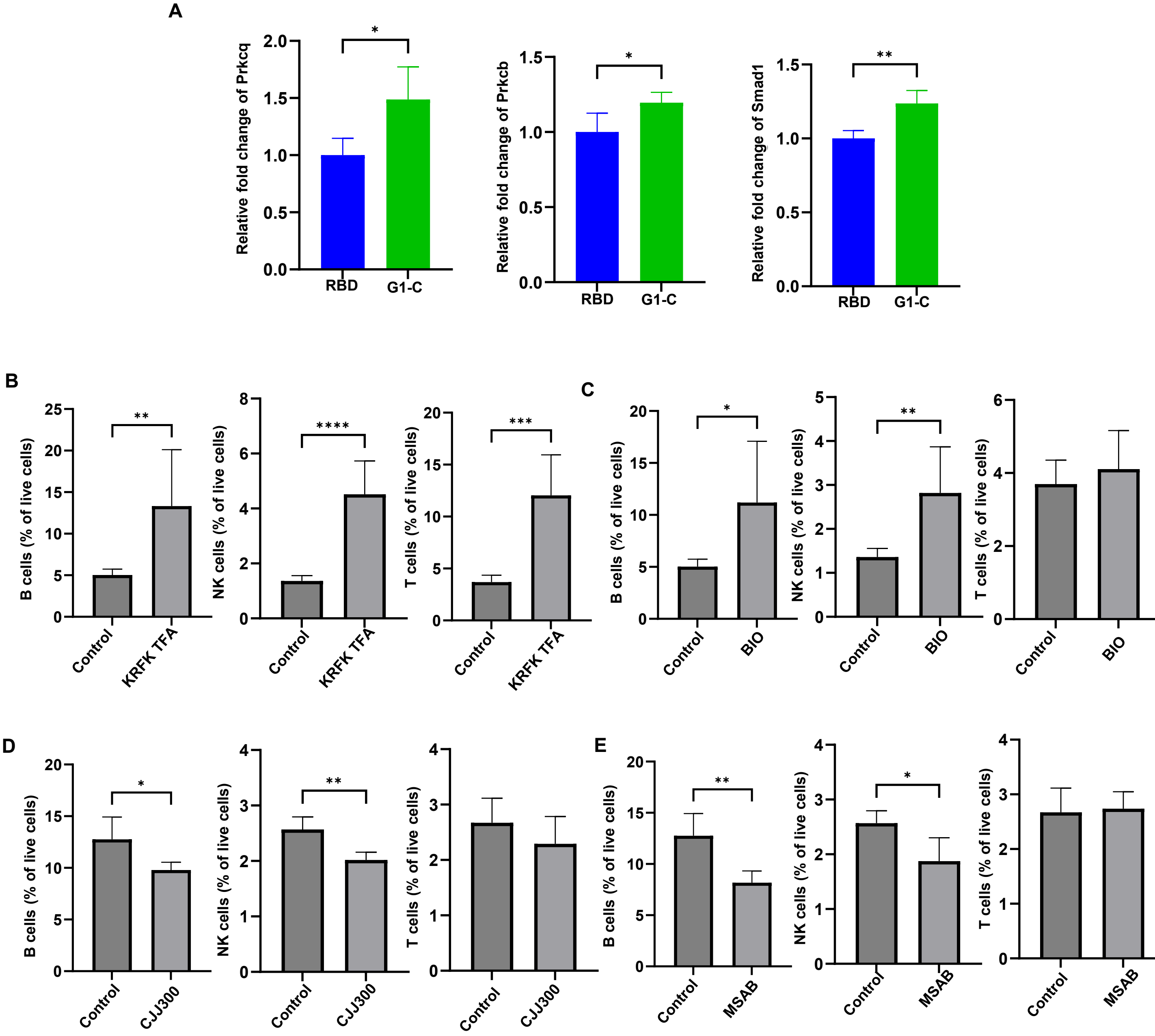

Supplementary Fig 7

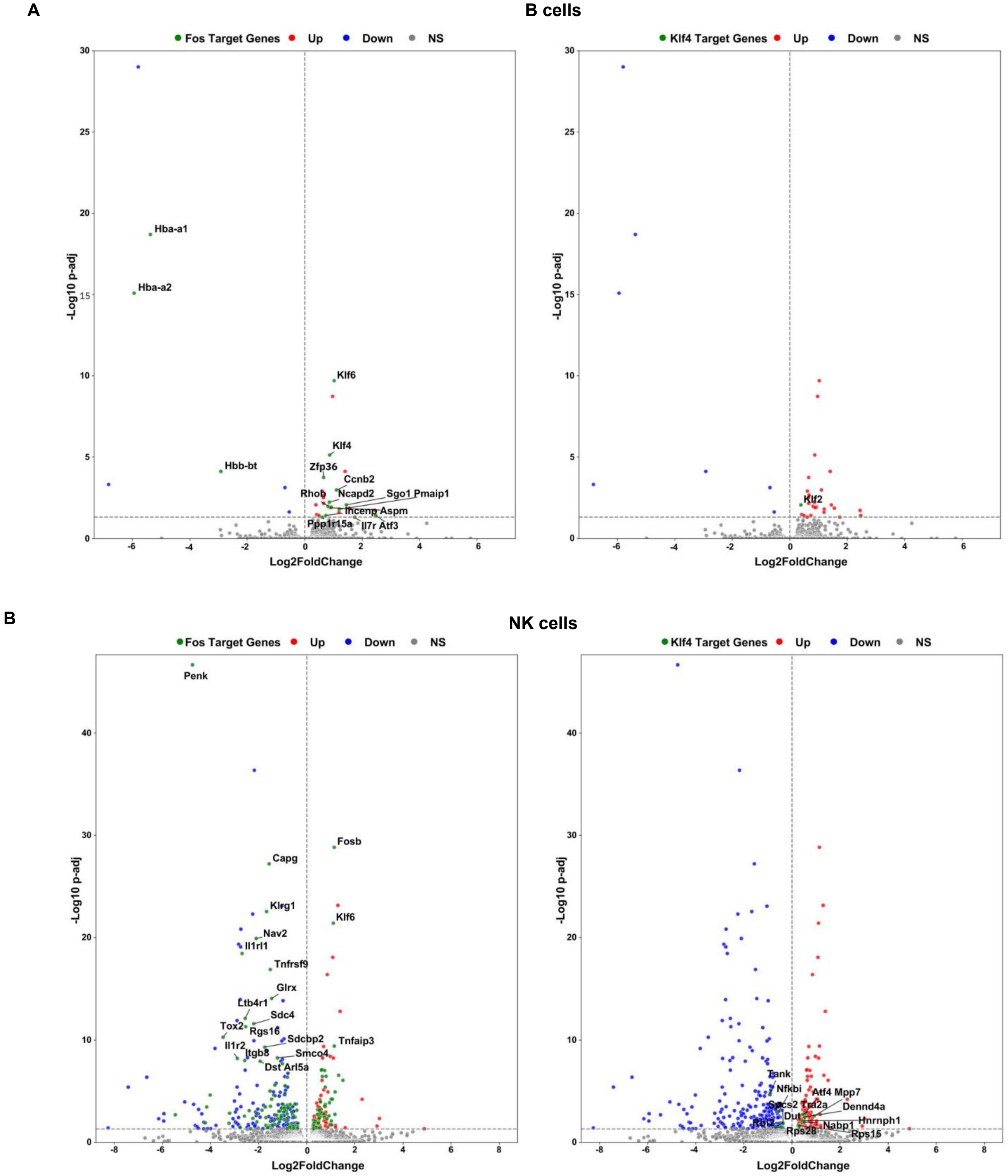

Supplementary Fig 8

A

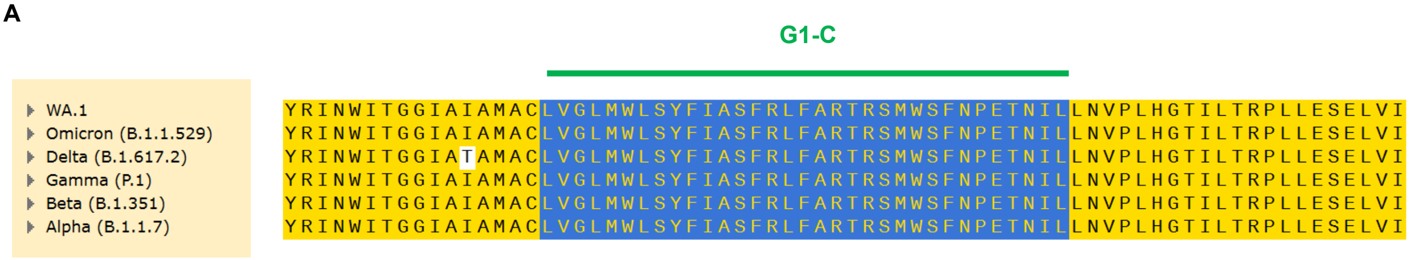

Supplementary Fig 9

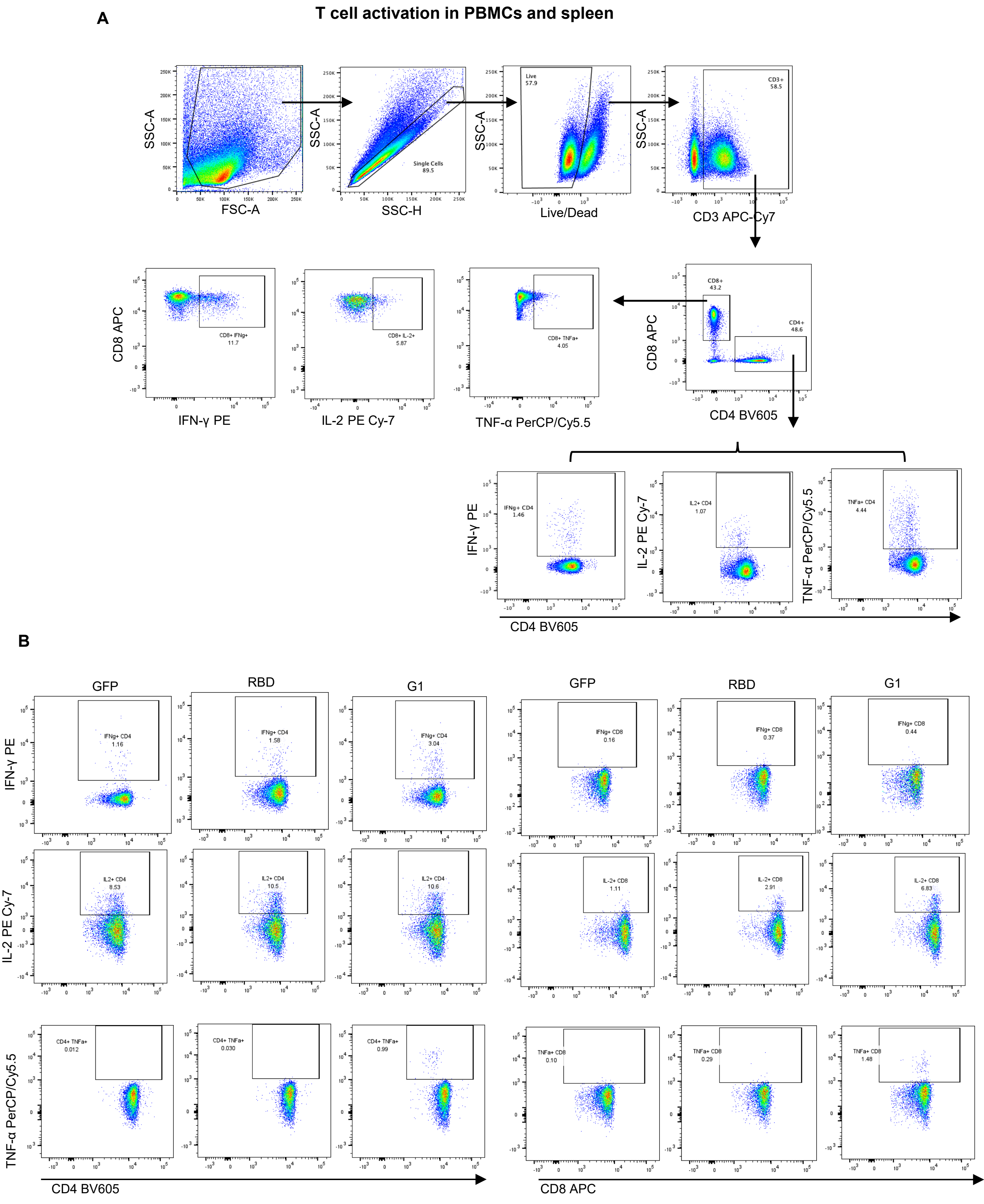

Supplementary Fig 10

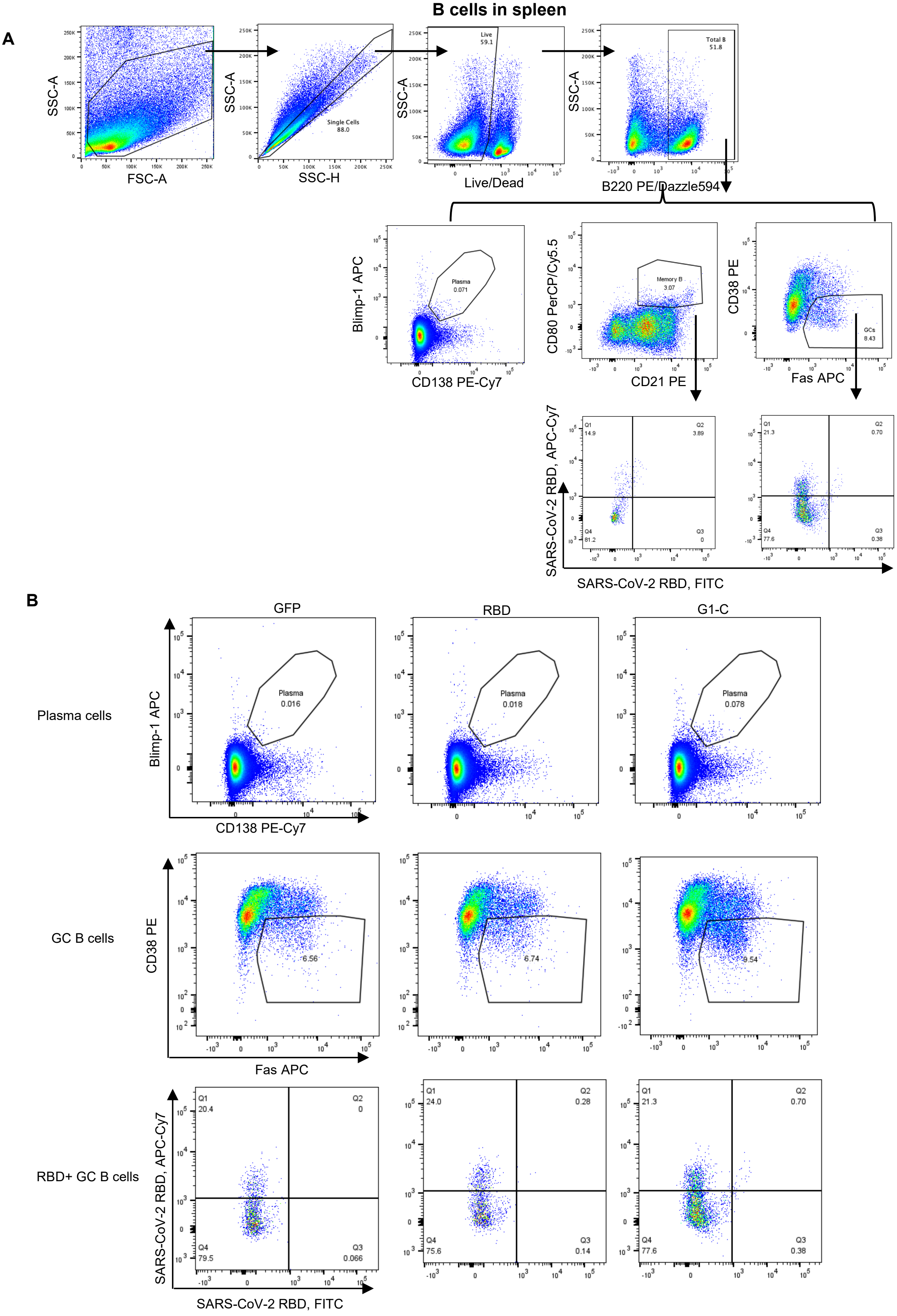

Supplementary Fig 11

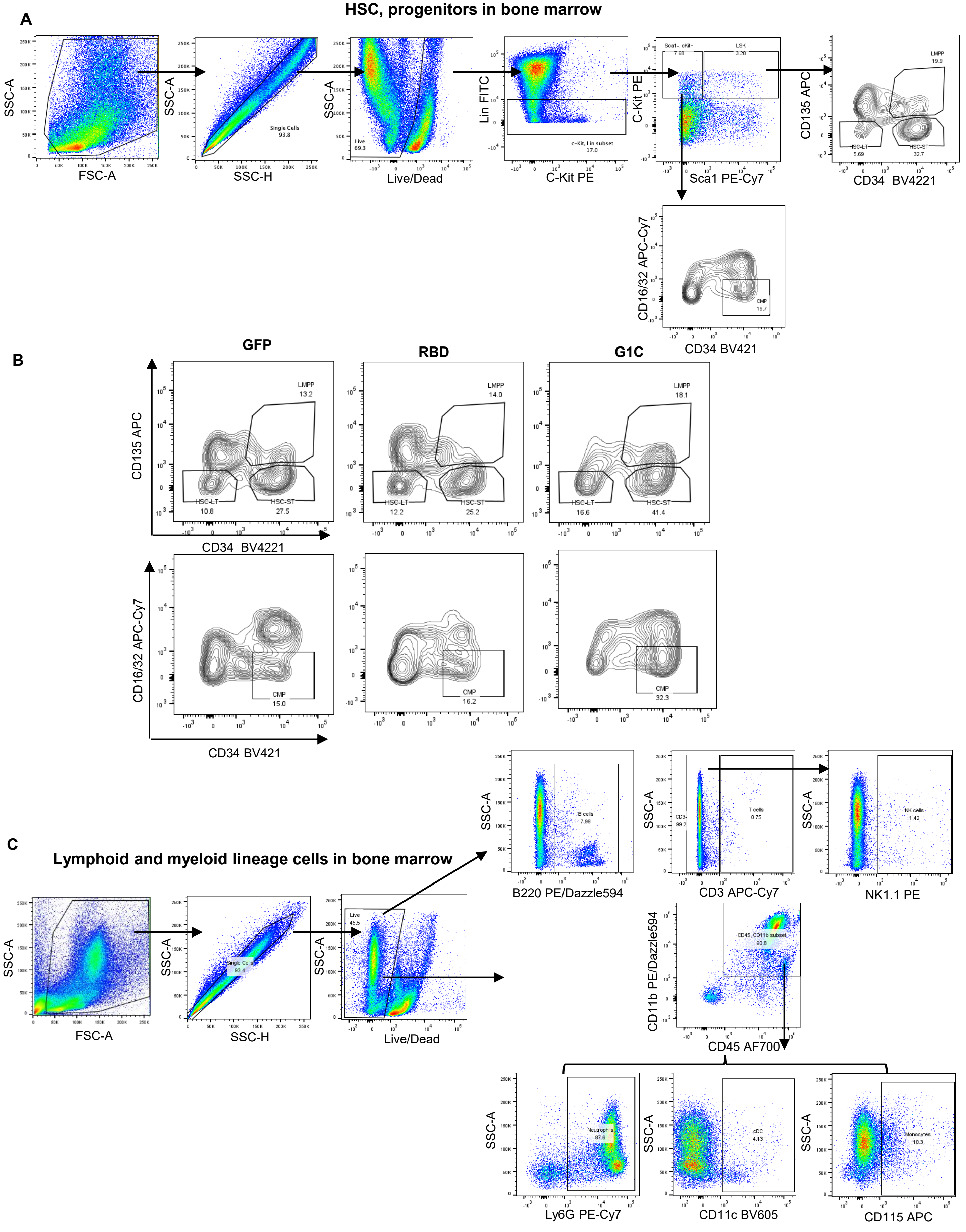

Supplementary Fig 12

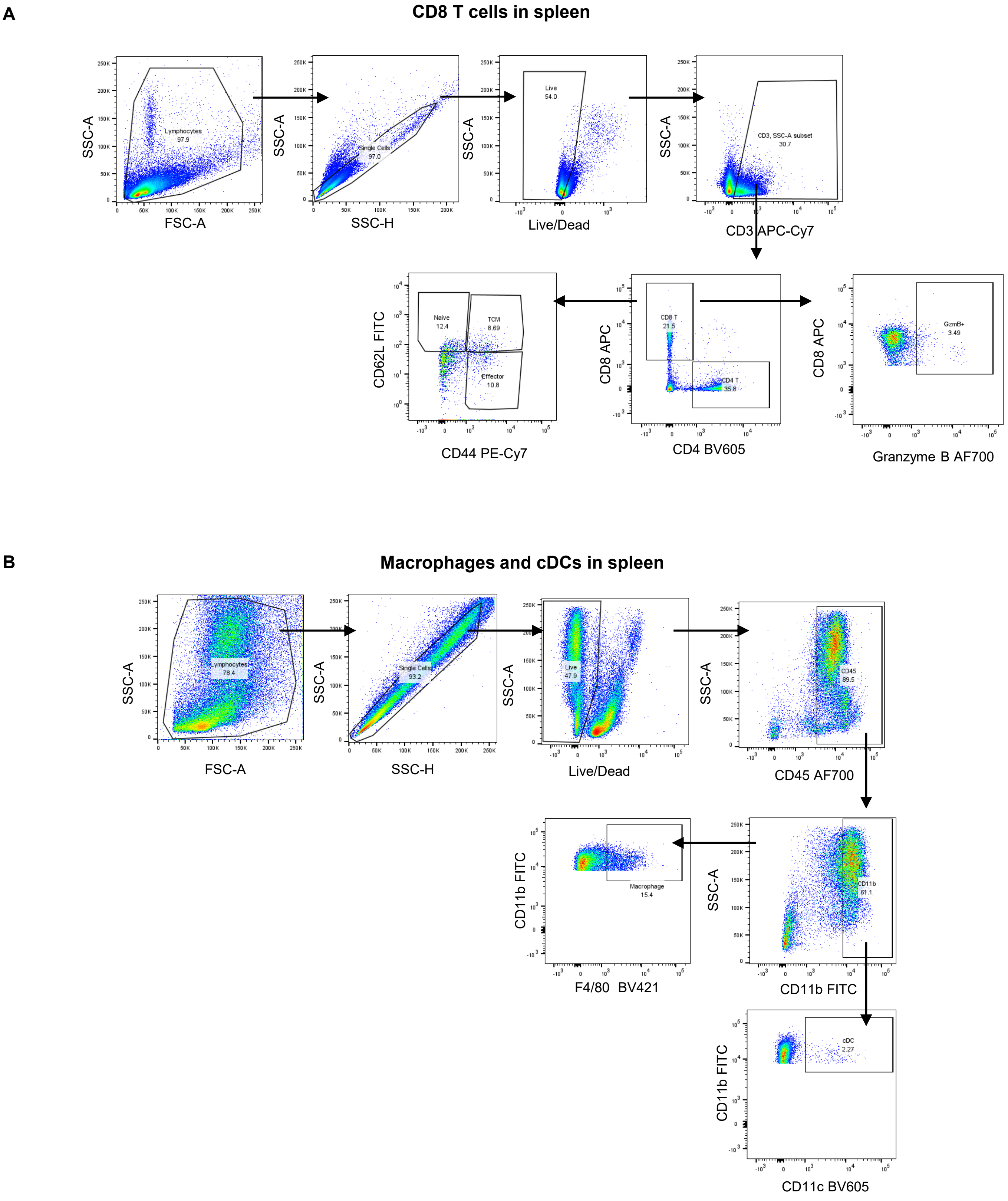

Supplementary Table 1

Antibody	Source	Cat. No.
Brilliant Violet 421™ anti-mouse CD34 Antibody	Biolegend	152208
Brilliant Violet 605™ anti-mouse CD41 Antibody	Biolegend	133921
FITC anti-mouse Lineage Cocktail	Biolegend	133302
PE anti-mouse c-Kit Antibody	Biolegend	105808
PerCP/Cyanine5.5 anti-mouse CD150 Antibody	Biolegend	115922
PE/Cyanine7 anti-mouse Sca-1 Antibody	Biolegend	122514
APC anti-mouse CD135 Antibody	Biolegend	135310
APC/Cyanine7 anti-mouse CD16/32 Antibody	Biolegend	101328
Brilliant Violet 421™ anti-mouse F4/80 Antibody	Biolegend	123137
Brilliant Violet 605™ anti-mouse CD11c Antibody	Biolegend	117334
FITC anti-mouse CD3 Antibody	Biolegend	100204
FITC anti-mouse B220 Antibody	Biolegend	103206
FITC anti-mouse NK1.1 Antibody	Biolegend	156508
PE anti-mouse Siglec-F Antibody	Biolegend	155506
FITC anti-mouse/human CD11b Antibody,	Biolegned	101206
PE/Cyanine7 anti-mouse Ly-6G Antibody	Biolegend	127618
APC anti-mouse CD115 Antibody	Biolegend	135510
Alexa Fluor® 700 anti-mouse CD45 Antibody	Biolegend	103128
Brilliant Violet 605™ anti-mouse CD4 Antibody	Biolegend	100451
PE anti-mouse NK-1.1 Antibody	Biolegend	108708
PE/Dazzle™ 594 anti-mouse/human CD45R/B220 Antibody	Biolegend	103258
APC anti-mouse CD8a Antibody	Biolegend	100712
APC/Cyanine7 anti-mouse CD3ε Antibody	Biolegend	100330
Brilliant Violet 605™ anti-mouse CD4 Antibody	Biolegend	100451
IFN gamma Monoclonal Antibody (XMG1.2), PE	(eBioscience	12-7311-82
PerCP/Cyanine5.5 anti-mouse TNF-α Antibody	Biolegend	506322
PE/Cyanine7 anti-mouse IL-2 Antibody	Biolegend	503832
FITC anti-mouse CD21/CD35 Antibody	Biolegend	123408
PE anti-mouse CD38 Antibody	Biolegend	102708
PE/Cyanine7 anti-mouse CD138 (Syndecan-1) Antibody	Biolegend	142514
APC anti-mouse CD95 (Fas) Antibody	Biolegend	152604
Alexa Fluor® 700 anti-mouse CD23 Antibody	Biolegend	101632
PerCP/Cyanine5.5 anti-mouse CD80 Antibody	Biolegend	104722
Alexa Fluor® 700 anti-human/mouse Granzyme B Antibody	Biolegend	396426
FITC anti-mouse CD62L Antibody	Biolegend	104406
PE/Cyanine7 anti-mouse/human CD44 Antibody	Biolegend	103030
APC anti-mouse Blimp-1 Antibody	Biolegend	150007

Supplementary Table 2

Cell type	GFP1	GFP2	GFP3	RBD1	RBD2	RBD3	G1-C1	G1-C2	G1-C3
B cells	265	411	382	837	690	358	713	947	1345
Dendritic cells	688	456	460	1189	2236	1013	474	1161	1266
Eo/Baso prog.	18	23	27	34	24	12	96	80	33
Ery/Mk prog.	2	1	6	0	5	2	28	12	9
Erythroblasts	3	11	3	3	2	23	0	7	0
Gran/Mono prog.	7	10	12	17	5	8	68	90	48
LMPPs	44	40	63	56	54	43	220	200	128
Mk prog.	0	0	5	2	1	3	3	4	3
Mono prog.	6	11	6	16	9	3	77	89	49
Monocytes	1276	1902	1608	1823	2870	1367	3269	4982	2890
Neutro prog.	3	6	17	11	8	2	95	138	70
Neutrophils	7800	8501	11595	6465	5862	5125	6451	2351	3633
NK cells	236	229	471	201	314	282	583	1207	988
pre-B	297	1367	617	2401	1218	504	1710	3382	2310
T cells	14	12	25	19	16	25	27	24	34
Total	10659	12980	15297	13074	13314	8770	13814	14674	12806

Supplementary Figure 1. Expression of mRNA constructs and characterization of mRNA-lipid nanoparticles (LNPs)

(A) Western blot analysis of expression of all 24 mRNA constructs in HEK293T cells. Cells (1×10^5) were transfected with 500 ng mRNA and collected for analysis 24 hrs later. Blots were probed with anti-SARS-CoV-2 Spike protein and anti-GAPDH. Grey intensity of the Western blotting strips was analyzed with software of Image J.

(B) Particle size and zeta potential of mRNA-LNPs, as determined by dynamic light scattering (DLS).

Supplementary Figure 2. Analysis of neutralizing activity and memory T cell responses in spleen of mice immunized with G1 to G6 vaccines

(A) XBB.1.5-RBD specific IgG titers in plasma, as quantified by ELISA.

(B) Neutralizing activity of plasma from mice immunized with indicated vaccines against Beta and Delta SARS-CoV-2 pseudoviruses. Mouse plasma was diluted 2500-fold for the assay (n=5).

(C, D) Spike-specific $CD4^+$ (B) and $CD8^+$ (C) T cell responses of mice immunized with indicated pooled vaccines, as measured in spleen and based on intracellular IFN-γ^+, IL-2^+ or TNFα^+ staining. Asterisks represent statistical significance (*$p < 0.05$; **$p < 0.01$; ***$p < 0.001$; ****$p < 0.0001$) as determined by unpaired t-test (A) and one-way ANOVA (B, C). Error bars indicate standard error of the mean (SEM).

Supplementary Figure 3. Immunization with G1-C mRNA vaccine promotes potent neutralizing activity and induces membrane-specific memory T cell responses in spleen

(A) Neutralizing activity in plasma of mice immunized with indicated vaccines against WA1, Beta, Delta and Omicron SARS-CoV-2 pseudoviruses. Plasma was diluted 2500-fold for the assay.

(B, C) Membrane-specific $CD4^+$ (B) and $CD8^+$ (C) T cell responses seen following immunization with indicated vaccines, measured in spleen by intracellular staining of IFN-γ^+, IL-2^+ and TNFα^+.

Asterisks represent statistical significance (*$p < 0.05$; **$p < 0.01$; ***$p < 0.001$; ****$p < 0.0001$) as determined by unpaired t-test (A) and one-way ANOVA (B, C). Error bars indicate standard error of the mean (SEM).

Supplementary Figure 4. Immunization with G1-C mRNA vaccine promotes CD8 T cell and Classical Dendritic Cells (cDCs) responses in spleen

(A) Analysis of the proportion of cytotoxic, naïve, central memory and effector CD8 T cells in spleen of mice immunized with indicated vaccines, based on flow cytometry (n=5).

(B) Analysis of the proportion of antigen-presenting cells including macrophages and cDCs in spleen of mice immunized with indicated vaccines, based on flow cytometry (n=5).

Asterisks represent statistical significance (*$p < 0.05$; **$p < 0.01$; ***$p < 0.001$; ****$p < 0.0001$) as determined by one-way ANOVA. Error bars indicate standard error of the mean (SEM).

Supplementary Figure 5. Identification of bone marrow cell types by scRNAseq

(A) The expression levels of 3 cell markers for each cell type are visualized in dot plots, related to Fig 4C.

(B) Expression of markers of progenitor cells highlighted on UMAP, related to Fig 4E.

Supplementary Figure 6. TGF-β and Wnt signaling pathways regulate B and NK cell differentiation in bone marrow

(A) RT-PCR analysis of expression of *Smad1*, *Prkcb* and *Prkcq* transcripts in BM cells ($5×10^6$) of mice immunized with indicated vaccines. Values are normalized to Gapdh; shown is the relative fold-change (n=4).

(B) The proportion of living B, NK and T cells seen after treatment (48 hrs) of fresh isolated mouse BM cells ($2×10^6$) with KRFK TFA (100 μM), a TGF-β agonist. Cells were analyzed by flow cytometry (n=7).

(C) The proportion of living B, NK and T cells seen after treatment (48 hrs) of fresh isolated mouse BM cells (2×10^6) with BIO (1 μM), a Wnt/β-catenin signaling agonist. Cells were analyzed by flow cytometry (n=7).

(D) The proportion of living B, NK and T cells seen after treatment (48 hrs) of fresh isolated mouse BM cells (2×10^6) with CJJ300 (10 μM), a TGF-β inhibitor. Cells were analyzed by flow cytometry (n=5).

(E) The proportion of living B, NK and T cells seen after treatment (48 hrs) of fresh isolated mouse BM cells (2×10^6) with MSAB (4 μM), a Wnt/β-catenin signaling inhibitor. Cells were analyzed by flow cytometry (n=5).

Asterisks represent statistical significance (*$p < 0.05$; **$p < 0.01$; ***$p < 0.001$; ****$p < 0.0001$) as determined by one-way ANOVA. Error bars indicate standard error of the mean (SEM).

Supplementary Figure 7. Fos or Klf4 target genes in B and NK cells.

(A) Volcano plots showing differential expression of Fos or Klf4 target genes in B cells from mice immunized with G1-C versus RBD vaccine, analyzed using DESeq2. ChIP-Atlas 3.0 was used identify Fos, Klf4, and Klf6 target genes at the TSS ± 5k from M. musculus (mm10). Red dots, upregulated genes; blue, downregulated genes; gray, non-significant changes. Green dots indicate DEGs overlapping in both Fos or Klf4 target genes.

(B) Volcano plots showing differential expression of Fos or Klf4 target genes in NK cells from mice immunized with G1-C versus RBD vaccine, analyzed using DESeq2. ChIP-Atlas 3.0 was used identify Fos and Klf4 target genes at the TSS ± 5k from M. musculus (mm10). Red dots, upregulated genes; blue, downregulated genes; gray, non-significant changes. Green dots indicate DEGs overlapping in both Fos or Klf4 target genes.

Supplementary Figure 8. G1-C sequence alignment

(A) Sequence alignment of G1-C epitope from multiple SARS-CoV-2 variants.

Supplementary Figure 9. Flow cytometry gating strategy for T cell responses in PBMCs and spleen

(A) Spike-specific T cell gating strategy. Spike-specific CD4$^+$ (CD3$^+$CD4$^+$IFN-γ$^+$/IL-2$^+$/TNFα$^+$) and CD8$^+$ (CD3$^+$CD8$^+$IFN-γ$^+$/IL-2$^+$/TNFα$^+$) T cells were gated from the CD3$^+$ population after removing doublets and dead cells.

(B) Representative flow cytometry plots of IFN-γ$^+$/IL-2$^+$/TNFα$^+$ CD4$^+$/CD8$^+$ T cells in PBMCs.

Supplementary Figure 10. Flow cytometry gating strategy for B cells in spleen.

(A) Memory B cell, germinal center B cell, and plasma cell gating strategy. Memory B (B220$^+$CD21$^+$CD80$^+$), germinal center B (B220$^+$FAS$^+$CD38^{low}), and plasma (Blimp-1$^+$CD138$^+$) cells were gated after removing doublets and dead cells. RBD-binding memory B and germinal center B cells were identified by RBD proteins. The RBD-specific B cells could recognize RBD antigen and bind to RBD[53].

(B) Representative flow cytometry plots of plasma cells, germinal center B cells and RBD$^+$ germinal center B cells.

Supplementary Figure 11. Flow cytometry gating strategy for immune cells in bone marrow.

(A) LT-HSC, ST-HSC, LMPP, and CMP gating strategy. LT-HSCs (Lin$^-$Sca1$^+$c-Kit$^+$CD34$^-$CD135$^-$), ST-HSCs (Lin$^-$Sca1$^+$c-Kit$^+$CD34$^+$CD135$^-$), LMPPs (Lin$^-$Sca1$^+$c-Kit$^+$CD34$^+$CD135$^+$), and CMPs (Lin$^-$Sca1$^-$c-Kit$^+$CD34$^+$CD16/32$^-$) were gated after removing doublets and dead cells.

(B) Representative flow cytometry plots of LT-HSC, ST-HSC, LMPP, and CMP.

(C) Lymphoid and myeloid lineage cell gating strategy. Lymphoid lineage cells included B (B220$^+$), NK (CD3$^-$NK1.1$^+$), and T (CD3$^+$) cells. Myeloid lineage cells included neutrophils (CD45$^+$CD11b$^+$Ly6G$^+$), cDCs (CD45$^+$CD11b$^+$CD11c$^+$), and monocytes (CD45$^+$CD11b$^+$CD115$^+$). All cells were gated after removing doublets and dead cells.

Supplementary Figure 12. Flow cytometry gating strategy for CD8 T cells subsets and APCs in spleen.

(A) Granzyme B, naïve, central memory and effector CD8 T gating strategy. Granzyme B^+ $CD8^+$ T cells ($CD3^+CD8^+$Granzyme B^+), naïve $CD8^+$ T cells ($CD3^+CD8^+CD62L^+CD44^-$), central memory $CD8^+$ T cells ($CD3^+CD8^+CD62L^+CD44^+$), and effector $CD8^+$ T cells ($CD3^+CD8^+CD62L^-CD44^+$) were gated after removing doublets and dead cells.

(B) Antigen-presenting cells gating strategy. Macrophages ($CD45^+CD11b^+F4/80^+$) and cDCs ($CD45^+CD11b^+CD11c^+$) were gated after removing doublets and dead cells.
